# Mucosal-associated invariant T cells are associated with insulin resistance in childhood obesity, and disrupt insulin signalling via IL-17

**DOI:** 10.1007/s00125-022-05682-w

**Published:** 2022-03-19

**Authors:** Ronan Bergin, David Kinlen, Nidhi Kedia-Mehta, Eadaoin Hayes, Féaron C. Cassidy, Declan Cody, Donal O’Shea, Andrew E. Hogan

**Affiliations:** 1 Kathleen Lonsdale Institute for Human Health Research, Maynooth University, Maynooth, County Kildare Ireland; 2 National Children’s Research Centre, Dublin, Ireland; 3 St Vincent’s University Hospital and University College Dublin, Dublin, Ireland

**Keywords:** Insulin resistance, Interleukin-17, Mucosal-associated invariant T cells, Obesity

## Abstract

**Aims/hypothesis:**

Mucosal-associated invariant T cells (MAIT cells) are an abundant population of innate T cells. When activated, MAIT cells rapidly produce a range of cytokines, including IFNγ, TNF-α and IL-17. Several studies have implicated MAIT cells in the development of metabolic dysfunction, but the mechanisms through which this occurs are not fully understood. We hypothesised that MAIT cells are associated with insulin resistance in children with obesity, and affect insulin signalling through their production of IL-17.

**Methods:**

In a cross-sectional observational study, we investigated MAIT cell cytokine profiles in a cohort of 30 children with obesity and 30 healthy control participants, of similar age, using flow cytometry. We then used a cell-based model to determine the direct effect of MAIT cells and IL-17 on insulin signalling and glucose uptake.

**Results:**

Children with obesity display increased MAIT cell frequencies (2.2% vs 2.8%, *p*=0.047), and, once activated, these produced elevated levels of both TNF-α (39% vs 28%, *p*=0.03) and IL-17 (1.25% vs 0.5%, *p*=0.008). The IL-17-producing MAIT cells were associated with an elevated HOMA-IR (*r*=0.65, *p*=0.001). The MAIT cell secretome from adults with obesity resulted in reduced glucose uptake when compared with the secretome from healthy adult control (1.31 vs 0.96, *p*=0.0002), a defect that could be blocked by neutralising IL-17. Finally, we demonstrated that recombinant IL-17 blocked insulin-mediated glucose uptake via inhibition of phosphorylated Akt and extracellular signal-regulated kinase.

**Conclusions/interpretations:**

Collectively, these studies provide further support for the role of MAIT cells in the development of metabolic dysfunction, and suggest that an IL-17-mediated effect on intracellular insulin signalling is responsible.

**Graphical abstract:**

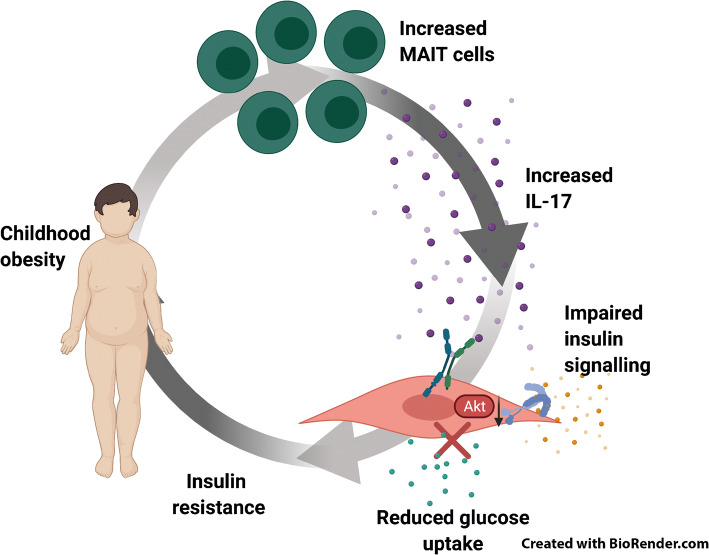

**Supplementary Information:**

The online version contains peer-reviewed but unedited supplementary material available at 10.1007/s00125-022-05682-w.

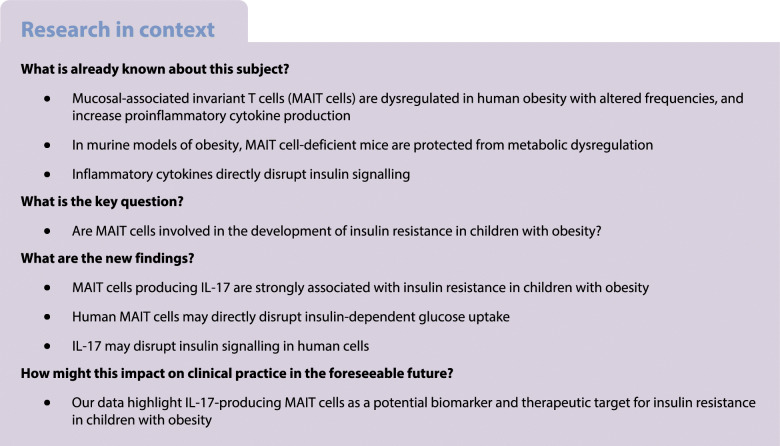



## Introduction

Mucosal-associated invariant T cells (MAIT cells) are a population of non-MHC restricted T cells that are important in the immune defence against bacterial and viral infections [1]. MAIT cells are early-responding T cells that are capable of rapidly producing multiple cytokines upon activation, such as IFNγ, TNF-α and IL-17 [2]. Dysregulated MAIT cell cytokine profiles have been reported in several diseases, including arthritis, cancer and inflammatory bowel disease [3]. MAIT cells have also been strongly implicated in the development and pathogenesis of obesity and its comorbid metabolic diseases. In both children and adults with obesity, MAIT cell frequencies and cytokine profiles are altered, with elevated IL-17 among the most prominent changes [4–6]. This phenotype was also observed in human obese adipose tissue [4, 5]. More recently Toubal et al provided more robust evidence that MAIT cells drive metabolic dysfunction by using MAIT cell-deficient murine models of obesity [7]. In these studies, mice deficient in MAIT cells were protected against weight gain and the development of metabolic dysfunction when fed a high-fat diet, and elevated IL-17 production by MAIT cells was noted across several sites [7]. However, there remains a relative paucity of data directly linking MAIT cells to metabolic dysfunction. We therefore investigated IL-17-producing MAIT cells in a cohort of children with obesity (CWO) and varying degrees of insulin resistance. We also assessed the direct impact of the MAIT cell secretome from adults with obesity (PWO) and IL-17 on insulin signalling.

## Methods

### Ethics statement and participants

Children’s Health Ireland Crumlin and Maynooth University Ethics Committee approved this study. Written informed consent/assent was obtained prior to partaking in the study. We enrolled a total of 80 children (50 children with obesity [CWO cohort] and 30 healthy control participants of similar age [HC cohort]; electronic supplementary material [ESM] Table 1) between 2016 and 2019. A fasting venous blood sample was obtained and used to assess fasting glucose/insulin levels together with MAIT cell frequencies and cytokine production. Inclusion criteria for the CWO cohort were classification as obese (>95th centile on the International Obesity Taskforce BMI centile charts [8]) and age between 7 and 17 years. Children with an underlying hormone deficiency, genetic disorder, inflammatory conditions or occurrence of recent acute infection were excluded. Fifty percent of the CWO cohort and 30% of the HC cohort were female. For studies investigating the impact of the MAIT cell secretome on glucose uptake, we investigated a previously reported cohort of 11 adults with obesity (PWO cohort) and 10 healthy adult controls [9].

### Preparation of peripheral blood mononuclear cells and flow cytometric analysis

**Fig. 1:**
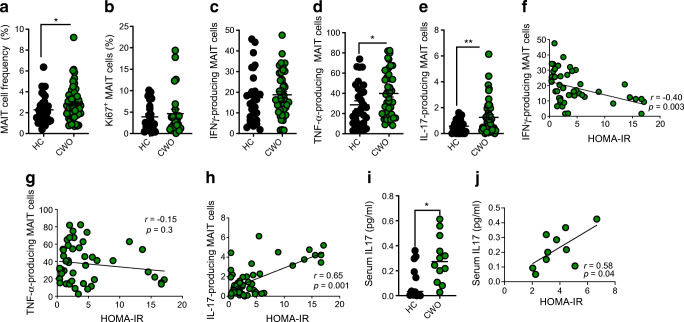
MAIT cells are altered in children with obesity and associated with insulin resistance. (**a**) Frequencies of MAIT cells (as a percentage of CD3^+^ T cells) in CWO and HC cohorts (*n* = 50 CWO; *n* = 30 HC). (**b**) Frequencies of Ki67^+^ MAIT cells (as a percentage of total MAIT cells) in HC and CWO. (**c**–**e**) Frequencies of MAIT cells producing (**c**) IFNγ, (**d**) TNF-α or (**e**) IL-17 in cohorts of HC or CWO (after stimulation with Dynabeads for 18 h). (**f**–**h**) Scatter graphs plotting frequencies of cytokine-producing MAIT cells (either IFNγ, TNF-α or IL-17) against HOMA-IR scores in CWO (*n* = 50). (**i**) Levels of serum IL-17 in HC and CWO cohorts. (**j**) Scatter graph plotting serum IL-17 levels against HOMA-IR scores in CWO (*n* = 10). Data are means ± SEM. *p<0.05; **p<0.01

Peripheral blood mononuclear cells were isolated by density centrifugation over Ficoll (Stemcell Technologies, Canada) from fresh peripheral blood samples. MAIT cell staining was performed using specific surface monoclonal antibodies namely CD3, CD161, CD8, Ki67 and TCRVα7.2 (all Miltenyi Biotec, Germany). Cell populations were acquired using an Attune NxT flow cytometer (Life Technologies, USA), and analysed using FlowJo software (Tree Star, USA). Results are expressed as a percentage of the parent population as indicated (Fig. 1a–e), and determined using unstained controls and flow minus-1 (FMO) controls.

### Analysis of cytokine production by MAIT cells

Peripheral blood mononuclear cells (1 x 10^6^) were stimulated using TCR activation beads (anti-CD3/CD28 Dynabeads) (Thermo Fisher Scientific, USA) in complete RPMI medium containing charcoal-stripped FBS (Gibco, USA) for 18 h in the presence of a protein transport inhibitor (BioLegend, USA). After 18 h, intracellular cytokine staining (IFNγ, TNF-α and IL-17; all Miltenyi Biotech) was performed using a True-Nuclear staining kit (BioLegend). For investigations into the impact of the MAIT cell secretome, purified MAIT cells (1 x 10^6^) from either healthy adult controls (HAC cohort) or adults with obesity (PWO cohort) were stimulated as above (no protein transport inhibitor), and supernatants were harvested for ELISA (R&D Systems, USA) and glucose uptake assay.

### Analysis of plasma IL-17 levels

Plasma levels of IL-17 was measured using a high-sensitivity Quantikine ELISA kit from R&D Systems.

### Glucose uptake assay

Human skeletal muscle cells (HSMCs; 2.5 x 10^6^) (Sigma Aldrich, Germany) were switched to insulin-free medium for 24 h before treatment with insulin (100 μmol/l) (Sigma Aldrich) for 40 min in the absence or presence of MAIT cell culture supernatant (with the addition of 1 μg/ml IL-17 neutralising antibody [InvivoGen, USA] for 30 min for the blocking experiments) or recombinant cytokine (IL-17 [50 ng/ml] or TNF-α [10 ng/ml], both from BioLegend). Glucose uptake was measured using the Glucose Uptake-Glo assay (Promega, USA) according to the manufacturer’s protocol.

### Analysis of insulin signalling

HSMCs (2.5 × 10^6^) were cultured in six-well plates and treated with recombinant IL-17 (50/ng) for 2 h, followed by insulin (100 μmol/l) for the indicated times before harvesting for western blotting. Cells were lysed in NP-40 lysis buffer (50 mmol/l Tris-HCI, pH 7.4, containing 150 mmol/l NaCl, 1% w/v IGEPAL (Sigma Aldrich), and complete protease inhibitor mixture [Roche, Switzerland]). Samples were resolved using SDS-PAGE, and transferred to nitrocellulose membranes before analysis with anti-p-Akt, anti-p-ERK (extracellular signal-regulated kinase) (both Cell Signaling Technology, USA) or anti-β-actin (Sigma) antibodies. Protein bands were visualised using enhanced chemiluminescence. Quantitative analysis of western blot analysis was performed using ImageJ software (https://imagej.nih.gov/ij/download.html).

### Statistical analysis

Statistical analysis was performed using GraphPad Prism 6 software (https://www.graphpad.com/scientific-software/prism/). Data are expressed as means ± SEM. We determined differences between two groups using unpaired Student’s *t* test and the Mann–Whitney *U* test where appropriate. Analysis across three or more groups was performed using ANOVA. Correlations were determined using linear regression models and expressed using Pearson or Spearman’s rank correlation coefficient, as appropriate. Differences were considered significant at a *p* value <0.05.

## Results

The clinical characteristics of the cohorts are presented in ESM Table 1.

### MAIT cell frequencies and cytokine profiles are altered in children with obesity

We first investigated MAIT cell frequencies in the two cohorts, and found elevated MAIT cells frequencies in the CWO cohort compared with the HC cohort (Fig. 1a). We next investigated whether the increased frequencies of MAIT cells in the CWO group were due to proliferation, but did not observe any increase in Ki67 staining (Fig. 1b). Next we examined MAIT cell cytokine production after stimulation with anti-CD3/CD28 Dynabeads, and observed increased frequencies of both TNF-α- and IL-17-producing MAIT cells in the CWO group, but no difference in IFNγ-producing cells (Fig. 1c–e). Finally, we determined whether the observed cytokine profiles were associated with insulin resistance levels (as determined by HOMA-IR). IL-17-producing MAIT cells (but not IFNγ- or TNF-α-producing cells) were strongly associated with elevated HOMA-IR in CWO (Fig. 1f–h). To validate this observation, we measured plasma IL-17 levels, and observed elevated levels in CWO and a strong association with HOMA-IR (Fig. 1i, j).

### MAIT cells and IL-17 directly alter insulin-mediated glucose uptake by impairing Akt/ERK signalling

**Fig. 2:**
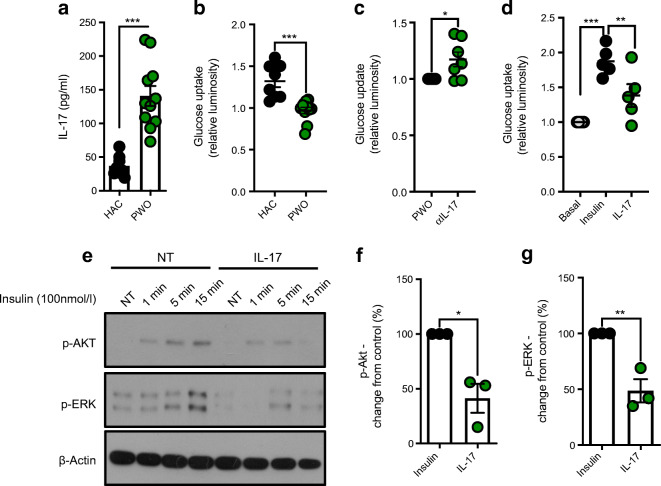
MAIT cells and IL-17 disrupt insulin-dependent glucose uptake. (**a**) Levels of IL-17 in supernatants of MAIT cells (after stimulation with Dynabeads for 18 h) from either the HAC or the PWO cohort (*n* = 8 HAC, *n* = 11 PWO). (**b**) Glucose uptake by HSMCs stimulated with insulin (100 nmol/l) for 15 min in the absence or presence of culture supernatants from (**a**) (*n* = 8 HAC, *n* = 11 PWO). (**c**) Glucose uptake by HSMCs stimulated with insulin (100 nmol/l) for 15 min in the presence of PWO cohort MAIT cell culture supernatants with or without treatment for 30 min of IL-17 neutralising antibody (αIL-17, 1 μg/ml) (*n* = 7). (**d**) Levels of glucose uptake by HSMCs alone (basal), or stimulated with insulin for 15 min in the absence or presence of recombinant human IL-17 (*n* = 5). (**e**) Representative western blot showing the effect of recombinant human IL-17 on insulin-mediated activation (as measured by phosphorylation) of Akt and ERK in HSMCs. (**f**, **g**) Per cent change (over insulin-treated controls) in p-Akt levels (**f**) or p-ERK levels (**g**) in HSMCs treated with IL-17 for 15 min, as measured by densitometry (*n* = 3). Data are means ± SEM. *p<0.05; **p<0.01; ***p<0.001. NT, no treatment

To determine whether MAIT cells directly affect insulin-mediated glucose uptake, we established a cell-based model using HSMCs. MAIT cell supernatants from the PWO cohort had elevated levels of IL-17 (Fig. 2a), with diminished insulin-mediated glucose uptake compared with the HAC cohort (Fig. 2b). Addition of a IL-17 neutralising antibody to supernatants from PWO increased glucose uptake (Fig. 2c). We next investigated the effect of recombinant IL-17 on insulin-mediated glucose intake into HSMCs, and demonstrated that IL-17 strongly inhibited glucose uptake in our cell-based model (Fig. 2d). Finally, we demonstrated that IL-17 inhibits the downstream signalling of insulin, with reduced expression of p-Akt and p-ERK (Fig. 2e–g).

## Discussion

MAIT cells are a subset of unconventional T cells that are capable of rapidly responding to stimulation, resulting in production of a range of cytokines [2]. This functional feature allows MAIT cells to play a critical role in host protection against invading pathogens and malignant cells. Numerous studies have reported dysregulated MAIT cell cytokine production in a range of human diseases, including obesity [10]. In adults with obesity, MAIT cell frequencies are markedly reduced, whereas we show that in childhood obesity they are elevated, but have previously demonstrated a rapid decrease with age in children with obesity [5]. In obesity, MAIT cells display elevated production of IL-17, with or without additional stimulation, and have been associated with the development of metabolic diseases [5–7].

In the current study, we demonstrate a strong association between IL-17-producing MAIT cells and HOMA-IR in a cohort of children with obesity. This mirrors a strong association between plasma IL-17 levels and HOMA-IR. Our data support the recent observation of improved glucose handling in mice deficient in MAIT cells, while mice with ‘human levels’ of MAIT cells (>10-fold increase) demonstrated poor glucose handling compared with their wild-type littermates [7]. A 1993 study by Hotamisligil et al demonstrated that TNF-α directly drives insulin resistance and disrupts insulin signalling [11]. In our analysis, IL-17 displayed a stronger association than TNF-α with the degree of insulin resistance in our cohort of children with obesity, and thus was the focus of our study.

Despite the strong association, the mechanisms through which MAIT cells affect glucose handling remain unclear. In our cell-based model, we have shown that MAIT cell supernatants from adults with obesity (which contained higher levels of IL-17) limited insulin-mediated glucose uptake into human muscle cells when compared with supernatants from healthy control MAIT cells. Furthermore, our studies show that IL-17 alone directly disrupts insulin-mediated glucose uptake in HSMCs. Previous studies using murine cells have demonstrated that IL-17 disrupts glucose uptake in hepatocytes and adipocytes, which have identical insulin signalling pathways [12, 13]. Furthermore, a recent study by Teijeiro et al demonstrated that inhibition of IL-17 suppressed diet-induced obesity and the development of glycaemic dysregulation [14]. Collectively, our data support a role for MAIT cells in the development of metabolic dysfunction, and suggest that an IL-17-mediated effect on intracellular insulin signalling may be involved in driving this dysfunction, potentially highlighting a novel therapeutic target for insulin resistance.

## Supplementary information

ESM 1 (PDF 90 kb)

## Data Availability

The data generated in this study are available from the corresponding author upon reasonable request.

## Abbreviations

CWO: Children with obesity (cohort)
ERK: Extracellular signal-regulated kinase
HAC: Healthy adult control (cohort)
HC: Healthy control (cohort)
HSMC: Human skeletal muscle cell
MAIT cells: Mucosal-associated invariant T cells
PWO: People with obesity (cohort)

## References

[1] Godfrey DI, Koay HF, McCluskey J, Gherardin NA (2019). The biology and functional importance of MAIT cells. Nat Immunol.

[2] Toubal A, Nel I, Lotersztajn S, Lehuen A (2019) Mucosal-associated invariant T cells and disease. Nat Rev Immunol 19(10):643–657. 10.1038/s41577-019-0191-y 31308521

[3] Chiba A, Tajima R, Tomi C, Miyazaki Y, Yamamura T, Miyake S (2012). Mucosal-associated invariant T cells promote inflammation and exacerbate disease in murine models of arthritis. Arthritis Rheum.

[4] Magalhaes I, Pingris K, Poitou C, Bessoles S, Venteclef N, Kiaf B (2015). Mucosal-associated invariant T cell alterations in obese and type 2 diabetic patients. J Clin Invest.

[5] Carolan E, Tobin LM, Mangan BA, Corrigan M, Gaoatswe G, Byrne G (2015). Altered distribution and increased IL-17 production by mucosal-associated invariant T cells in adult and childhood obesity. J Immunol.

[6] Brien AO, Kedia-Mehta N, Tobin L, Veerapen N, Besra GS, Shea DO et al (2020) Targeting mitochondrial dysfunction in MAIT cells limits IL-17 production in obesity. Cell Mol Immunol 17(11):1193–1195. 10.1038/s41423-020-0375-1 PMC7784973 32107463

[7] Toubal A, Kiaf B, Beaudoin L, Cagninacci L, Rhimi M, Fruchet B (2020). Mucosal-associated invariant T cells promote inflammation and intestinal dysbiosis leading to metabolic dysfunction during obesity. Nat Commun.

[8] Cole TJ, Lobstein T (2012). Extended international (IOTF) body mass index cut-offs for thinness, overweight and obesity. Pediatr Obes.

[9] O'Brien A, Loftus RM, Pisarska MM (2019). Obesity reduces mTORC1 activity in mucosal-associated invariant T cells, driving defective metabolic and functional responses. J Immunol.

[10] Pisarska MM, Dunne MR, O'Shea D, Hogan AE (2020) Interleukin-17 producing mucosal associated invariant T cells - emerging players in chronic inflammatory diseases? Eur J Immunol 50(8):1098–1108. 10.1002/eji.202048645 32617963

[11] Hotamisligil GS, Shargill NS, Spiegelman BM (1993). Adipose expression of tumor necrosis factor-alpha: direct role in obesity-linked insulin resistance. Science..

[12] Fabbrini E, Cella M, McCartney SA, Fuchs A, Abumrad NA, Pietka TA (2013). Association between specific adipose tissue CD4+ T-cell populations and insulin resistance in obese individuals. Gastroenterology..

[13] Zuniga LA, Shen WJ, Joyce-Shaikh B, Pyatnova EA, Richards AG, Thom C (2010). IL-17 regulates adipogenesis, glucose homeostasis, and obesity. J Immunol.

[14] Teijeiro A, Garrido A, Ferre A, Perna C, Djouder N (2021). Inhibition of the IL-17A axis in adipocytes suppresses diet-induced obesity and metabolic disorders in mice. Nat Metab.

