# Supplementary material for: Mucosal-associated invariant T cells are associated with insulin resistance in childhood obesity, and disrupt insulin signalling via IL-17
Source: Diabetologia. 2022 Mar 19;65(6):1012–7. doi: 10.1007/s00125-022-05682-w (PMC9076704; doi:10.1007/s00125-022-05682-w)
Supplement: Supplementary file 1 — (PDF 90 kb) [file 125_2022_5682_MOESM1_ESM.pdf]

# ESM Table 1: Cohort Characteristics

| | Controls | Children with Obesity | P Value |
|---|---|---|---|
| **n** | 30 | 50 | - |
| **Age – years (Range)** | 12.5<br>(7-17) | 13.6<br>(7-17) | 0.09<br>- |
| **Sex - % female** | 30% | 50% | - |
| **BMI z-score** | -0.3 | 2.15 | <0.0001 |
| **HOMA-IR** | 0.9 | 4.4 | <0.0001 |
| **Fasting Insulin (pmol/L)** | 22.8 | 127.3 | <0.0001 |
| **Fasting glucose (mmol/L)** | 5.05 | 5.04 | 0.89 |
| **Medications** | 0 | 0 | - |
